# Sailing in Uncharted Waters: Carefully Navigating the Polio Endgame

**DOI:** 10.1371/journal.pmed.1002141

**Published:** 2016-10-04

**Authors:** Elizabeth Miller, T. Jacob John

**Affiliations:** 1 Immunisation Hepatitis and Blood Safety Department, Public Health England, London, United Kingdom; 2 Child Health Foundation, New Delhi, India; 3 Department of Clinical Virology, Christian Medical College, Vellore, India

## Abstract

In a Perspective linked to the research article by Isobel Blake and colleagues, Elizabeth Miller and T. Jacob John discuss the path towards global polio eradication and the challenges, strategies, and necessary precautions around oral polio vaccine cessation.

## Current Status of Global Polio Eradication

In 1988, the World Health Assembly (WHA) passed a historic resolution to eradicate polio by the year 2000, resulting in the creation of the largest public–private partnership for health—the Global Polio Eradication Initiative (GPEI). When GPEI was launched, wild polio virus (WPV) was endemic in 125 countries and resulted annually in the paralysis of more than 350,000 people, mainly children. By 2000, the number of endemic countries had been reduced to 20, with the last WPV type 2 case occurring in 1999 [1]. Interruption of transmission of WPV types 1 and 3 proved more difficult, with the last WPV3 case occurring in November 2012; WPV1 cases are still endemic in Pakistan and Afghanistan, where a total of 19 were reported in 2016 as of July 12th [2].

The key tool of the GPEI has been the trivalent oral polio vaccine (tOPV), which induces both humoral and mucosal immunity protecting against paralysis and gut infection, thereby limiting transmission and inducing herd immunity. As the immune response to type 2 in tOPV is dominant, monovalent vaccines containing types 1 or 3 and a bivalent 1+3 OPV (bOPV) were developed and used in supplementary immunisation activities from 2005 and 2009, respectively. These vaccines played a significant role in achieving interruption of transmission of types 1 and 3 in India, where poor immunogenicity of tOPV was a major factor in their continued circulation, and helped in Nigeria and Pakistan also [3]. However, their use, combined with low tOPV coverage, has led to low type 2 immunity in some populations, with the attendant risk of emergence of circulating vaccine-derived polio virus type 2 (cVDPV2). Through molecular techniques that had just become available, the phenomenon of cVDPVs was first identified in 2000 when analysis of type 1 isolates from an outbreak in Hispaniola showed them to be vaccine derived [4]. Subsequently, retrospective analysis of isolates from a type 2 outbreak in Egypt from 1988 to 1993 confirmed its origin as Sabin virus [4]. Since then, many cVDPV polio outbreaks have been identified, and, although they can occur with any of the three vaccine strains, over 90% are due to type 2 [5]. Moreover, type 2 vaccine virus accounts for 40% of sporadic vaccine-associated paralytic polio cases. With no WPV2 cases since 1999, continued use of Sabin type 2 strain, with its associated risks, has become difficult to justify.

## The Polio Eradication and Endgame Strategic Plan 2013–2018

In 2013, the GPEI launched its new Endgame Plan following the declaration by the WHA (May 2012) that ending polio was a “programmatic emergency for global public health” [6]. The expectation was WPV transmission would stop by end 2014 and new cVDPV outbreaks would be interrupted within 120 days of confirmation of the index case. Although the former has not been achieved, the removal of Nigeria from the list of polio-endemic countries in September 2015 was a major success. A second objective was replacement of tOPV with bOPV (tOPV to bOPV switch) in the routine schedule to remove risks associated with continued use of type 2 Sabin virus. However, loss of population immunity to type 2 runs the risk of emergence of cVDPV2s as the Sabin virus washes out from the population. In addition, there are longer-term risks of leakage of WPV2 from sites manufacturing the inactivated polio vaccine (IPV) from wild-type strains despite adherence to all the bio-safety practices stipulated in the WHO Global Action Plan version 3. Deliberate release is also a concern, as may have happened in India, where a laboratory WPV type 2 strain was detected in 2002 in children [3]. Long-term immunodeficient excretors of vaccine-derived poliovirus present a further potential hazard.

## Mitigating the Risks Associated with Removing Sabin Virus Type 2 from OPV

To mitigate these risks, a number of precautions have been put in place. First, the switch from tOPV to bOPV was globally synchronised in all 155 OPV-using countries and took place in the two weeks from April 16 to May 1, 2016. Second, a dose of IPV was recommended to be introduced in all 126 countries exclusively using OPV six months before the switch. This would have boosted humoral and mucosal immunity in previously OPV-immunised children and would maintain some background humoral immunity to reduce the risk of paralytic disease in the event of cVDPV2 emergence. Third, multiple campaigns with tOPV were conducted in the run-up to the switch, especially in high-risk areas such as Nigeria and Pakistan, to reduce the risk of development of new vaccine-derived polio virus (VDPV) 2 lineages and to interrupt any undetected lineages already in circulation. In the accompanying paper [7], Isobel Blake and colleagues nicely illustrate the correlation between low population immunity to type 2 and the risk of cVDPV2 cases in Nigeria and Pakistan; they also describe the encouraging increase in type 2 immunity that resulted from the tOPV campaigns conducted in these countries prior to the switch. Their immunity forecasts have provided confidence to the WHO Strategic Advisory Group of Experts that the proposed withdrawal of OPV2 in April 2016 should go ahead. Fourth, because VDPV circulation can remain silent for some time, the monitoring system created for WPV eradication, namely acute flaccid paralysis surveillance with virological screening of all cases, has been supplemented with testing of sewage for polioviruses for the early detection of any type 2 polio virus before it causes paralytic cases [8]. Finally, because continued use of IPV is likely in the post-eradication era, alternative polioviruses are being developed that are more bio-safe than WPV for manufacturing IPV, especially in developing country settings [9]. Despite all these precautions, WHO has nevertheless developed a type 2 outbreak response protocol that relies on the judicious use of monovalent OPV type 2 (mOPV2) supplemented by IPV [10].

## Early Experience with the Switch

Despite the major logistical problems, the synchronised global tOPV to bOPV switch was successfully completed on schedule and, as of July 2016, has been verified in almost all countries [11]. Less successful was the introduction of IPV due to unanticipated vaccine shortages resulting from problems of rapid up-scaling of production in one enormous demand-step. Prioritisation of available supplies to countries most at risk of a cVDPV2 emergence was therefore undertaken, while reserving some stock for outbreak response if needed [12]. By April 2016, IPV had been introduced in all high-risk countries and some at lower risk, though supplies to the latter were unfortunately interrupted after introduction, and re-instatement of stock and supply to the remaining countries is now expected in the 4th quarter of 2017. In India, giving two fractional (1/5th) doses of IPV intradermally has eased the IPV vaccine shortage; this approach was based on evidence that the immunogenicity of two intradermal fractional doses at 6 and 14 weeks was similar to what is expected from one full dose at 14 weeks [13].

The value of enhanced environmental surveillance has already been shown by the detection of a cVDPV2 in sewage, in addition to a cVDPV2 case in Nigeria, and detection of a VDPV2 strain in sewage in India in the post-switch period. An immediate response with mOPV-2 campaigns took place in Nigeria and with one fractional dose of IPV in India based on careful risk assessment (Michel Zaffran, Director GPEI, personal communication). Furthermore, the identification in Nigeria of two children with acute flaccid paralysis (AFP) due to WPV1 between May 29 and June 18, 2016 underlines the importance of sustaining high-quality surveillance as part of the eradication efforts [14].

We are sailing in unchartered waters with many risks, but with all possible precautions in place to mitigate these. Removal of Sabin type 2 from OPV was the first step towards global OPV cessation; bOPV will be discontinued following certification of eradication of all WPVs. Until all risks of OPV cessation are understood and mitigated, continued IPV coverage will be necessary.

## Abbreviations

AFP: acute flaccid paralysis
bOPV: bivalent 1+3 OPV
cVDPV2: circulating vaccine-derived polio virus type 2
GPEI: Global Polio Eradication Initiative
IPV: inactivated polio vaccine
mOPV2: monovalent OPV type 2
OPV: trivalent oral polio vaccine
VDPV: vaccine-derived polio virus
WHA: World Health Assembly
WPV: wild polio virus
